# A new istiodactylid pterosaur, *Lingyuanopterus camposi* gen. et sp. nov., from the Jiufotang Formation of western Liaoning, China

**DOI:** 10.7717/peerj.13819

**Published:** 2022-07-26

**Authors:** Yizhi Xu, Shunxing Jiang, Xiaolin Wang

**Affiliations:** 1 Key Laboratory of Vertebrate Evolution and Human Origins, Institute of Vertebrate Paleontology and Paleoanthropology, Chinese Academy of Sciences, Beijing, China; 2 College of Earth and Planetary Sciences, University of Chinese Academy of Sciences, Beijing, China; 3 CAS Center for Excellence in Life and Paleoenvironment, Beijing, China

**Keywords:** Istiodactylidae, Jehol Biota, Jiufotang Formation, Pterosauria, Phylogeny, Bromalites

## Abstract

The Istiodactylidae is a group of pterodactyloids characterised by large nasoantorbital fenestrae and labiolingually compressed teeth, with several records reported from the Early Cretaceous of northeastern China and western Europe. Here we report a new istiodactylid, *Lingyuanopterus camposi* gen. et sp. nov. from the Jiufotang Formation of Lingyuan, Liaoning, northeastern China. The holotype is represented by a near-complete skull, mandible and atlas-axis complex. It is distinguished from other istiodactylids by several characters, including two autapomorphies: short triangular tooth crowns with sharp mesial and distal carinae limited to the distal teeth, mandibular symphysis occupying approximately a quarter the mandible length. We also report the presence of helical jaw joints in istiodactylids, and provide a revised diagnosis of the clade Istiodactylidae, which includes five genera: *Istiodactylus*, *Liaoxipterus*, *Nurhachius*, *Luchibang* and *Lingyuanopterus*. Four pellets containing fish fragments were observed and are tentatively interpreted as bromalites of *Lingyuanopterus*. Although members of this clade possess similar skull morphologies, istiodactylids vary in terms of their dentition, with at least three forms from the Jiufotang Formation alone. This may represent different feeding strategies, and also indicate a similarity between the pterosaur assemblages of northeastern China and Britain during the Early Cretaceous.

## Introduction

The Istiodactylidae is a group of pterodactyloids known from the Early Cretaceous, with members reported from western Europe and northeastern China (Hone et al., 2020). They are unique among pterosaurs with their large nasoantorbital fenestrae and labiolingually compressed teeth (Witton, 2013). The first istiodactylid was reported from the Late Barremian-Early Aptian Vectis Formation (Isle of Wight, England; Kerth & Hailwood, 1988), and was originally designated *Ornithodesmus latidens* (Seeley, 1901). Subsequently this taxon was assigned to a new genus, *Istiodactylus*, and the only member of its family, the Istiodactylidae (Howse, Milner & Martill, 2001). Since then, another seven species of istiodactylid pterosaurs, *Liaoxipterus brachyognathus*, *Nurhachius ignaciobritoi*, *Istiodactylus sinensis*, *Longchengpterus zhaoi*, *Hongshanopterus lacustris*, *Nurhachius luei*, and *Luchibang xingzhe* have been reported from the Jehol Biota, northeastern China (Dong & Lü, 2005; Wang et al., 2005; Andres & Ji, 2006; Wang et al., 2006; Wang et al., 2008; Zhou et al., 2019; Hone et al., 2020). The majority of them were reported from the Jiufotang Formation, except *Luchibang* was from the Yixian Formation (Hone et al., 2020). However, *Longchengpterus zhaoi* was treated as a junior synonym of *N. ignaciobritoi* by some authors (Lü, Xu & Ji, 2008; Zhou et al., 2019). Moreover, *Liaoxipterus* was solely represented by an anatomically uninformative, incomplete lower jaw and was suggested to be located at different phylogenetic positions among researchers (Andres & Ji, 2008; Wang et al., 2008; Lü, Xu & Ji, 2008; Witton, 2012). The referral of *Hongshanopterus lacustris* to the Istiodactylidae has also been widely questioned. This is primarily due to its inconsistent placement within phylogenetic analyses, suggesting it is not an istiodactylid (Witton, 2012; Andres, Clark & Xu, 2014; Zhou et al., 2019; Kellner et al., 2019; Hone et al., 2020). The tooth fragments of istiodactylids have also been reported in the Lower Cretaceous Wessex Formation of Britain, Camarillas Formation and La Huérguina Formation of Spain (Kühne, 1966; Krebs, 1985; Sánchez-Hernández, Benton & Naish, 2007; Vullo et al., 2009; Sweetman & Martill, 2010). A dentary with teeth similar to istiodactylids has also been reported from the uppermost Morrison Formation of Wyoming (Bakker, 1998), but recent research suggests its dentition is different to all known pterosaurs and its taxonomic affinities remain unknown (McLain & Bakker, 2018). There has been a long debate on the diet of istiodactylids, but scavenging seems to be the best supported hypothesis (Howse, Milner & Martill, 2001; Ősi, 2011; Witton, 2012; Martill, 2014; Bestwick et al., 2020; Jiang et al., 2020).

Some related taxa, including *Haopterus gracilis*, *Linlongopterus jennyae*, and *Mimodactylus libanensis* have been reported from the Cretaceous deposits of Lebanon and the Jehol Biota, northeastern China (Wang & Lü, 2001; Rodrigues et al., 2015; Kellner et al., 2019), and combined with the Istiodactylidae, forming a more inclusive clade, the Istiodactyliformes (Kellner et al., 2019; Hone et al., 2020). The occurrence of istiodactyliforms extends from the Barremian (based on tooth fragments of istiodactylids, Kühne, 1966; Krebs, 1985; Sánchez-Hernández, Benton & Naish, 2007; Vullo et al., 2009; Sweetman & Martill, 2010) to the Cenomanian (*Mimodactylus*, Kellner et al., 2019), and possibly to the Turonian if *Lonchodraco giganteus* is included (Rodrigues & Kellner, 2013), which is an istiodactyliform based on the phylogenetic analysis of Richards, Stumkat & Salisbury (2021, Fig. 4).

Here we report a new istiodactylid, *Lingyuanopterus camposi* gen. et sp. nov., from the Jiufotang Formation of Lingyuan, Liaoning, northeastern China. This new record enhances our knowledge of the morphology and taxonomy of the Istiodactylidae, and provides new information on the feeding behavior of istiodactylids.

## Materials & Methods

Pterosaur specimen IVPP V 17940 described here is housed at the Institute of Vertebrate Paleontology and Paleoanthropology, Chinese Academy of Sciences (IVPP/CAS). Since some elements of the posterior region of the skull are crushed or missing, more details, including sutures that are difficult to delineate under the microscope and bones obscured by matrix, are observed using Micro X-ray computed laminography (Micro-CL) in the Key Laboratory of Vertebrate Evolution and Human Origins, IVPP. CL Image segmentation and visualization were performed using VGStudio 3.0 (Volume Graphics, Baden-Württemberg, Germany).

We carried out a phylogenetic analysis based on the data matrix of the primary phylogenetic analysis of Richards, Stumkat & Salisbury (2021), with *Lingyuanopterus camposi* and another four species added (*Linlongopterus jennyae* and *M. libanensis* with their coding from Kellner et al. (2019), *Luchibang xingzhe* with its coding from Hone et al. (2020), and *Lonchodectes compressirostris*). The analysis is conducted with the software TNT v 1.5 (Goloboff & Catalano, 2016), performed *via* traditional search, with the random seed = 1, 10,000 replicates, 100 trees to save per replication and collapsing trees after search (Holgado et al., 2019). The data matrix is available in the Supplemental Information.

The electronic version of this article in Portable Document Format (PDF) will represent a published work according to the International Commission on Zoological Nomenclature (ICZN), and hence the new names contained in the electronic version are effectively published under that Code from the electronic edition alone. This published work and the nomenclatural acts it contains have been registered in ZooBank, the online registration system for the ICZN. The ZooBank LSIDs (Life Science Identifiers) can be resolved and the associated information viewed through any standard web browser by appending the LSID to the prefix http://zoobank.org/. The LSID for this publication is: urn:lsid:zoobank.org:pub:57EAFE78-9764-4777-A9DF-328325419767. The online version of this work is archived and available from the following digital repositories: PeerJ, PubMed Central SCIE and CLOCKSS.

## Results

### Systematic paleontology

**Table:** 

Pterosauria Kaup, 1834
Pterodactyloidea Plieninger, 1901
Istiodactyliformes Kellner et al., 2019
Istiodactylidae Howse, Milner & Martill, 2001

**Emended diagnosis.** istiodactyliforms with palatal ridge more developed posteriorly on the palate (synapomorphy), a large nasoantorbital fenestra occupying more than 50% of the length of the skull anterior to the jaw articulation, teeth strongly labiolingually compressed and restricted to the anterior part of the skull.

**Figure 1:**
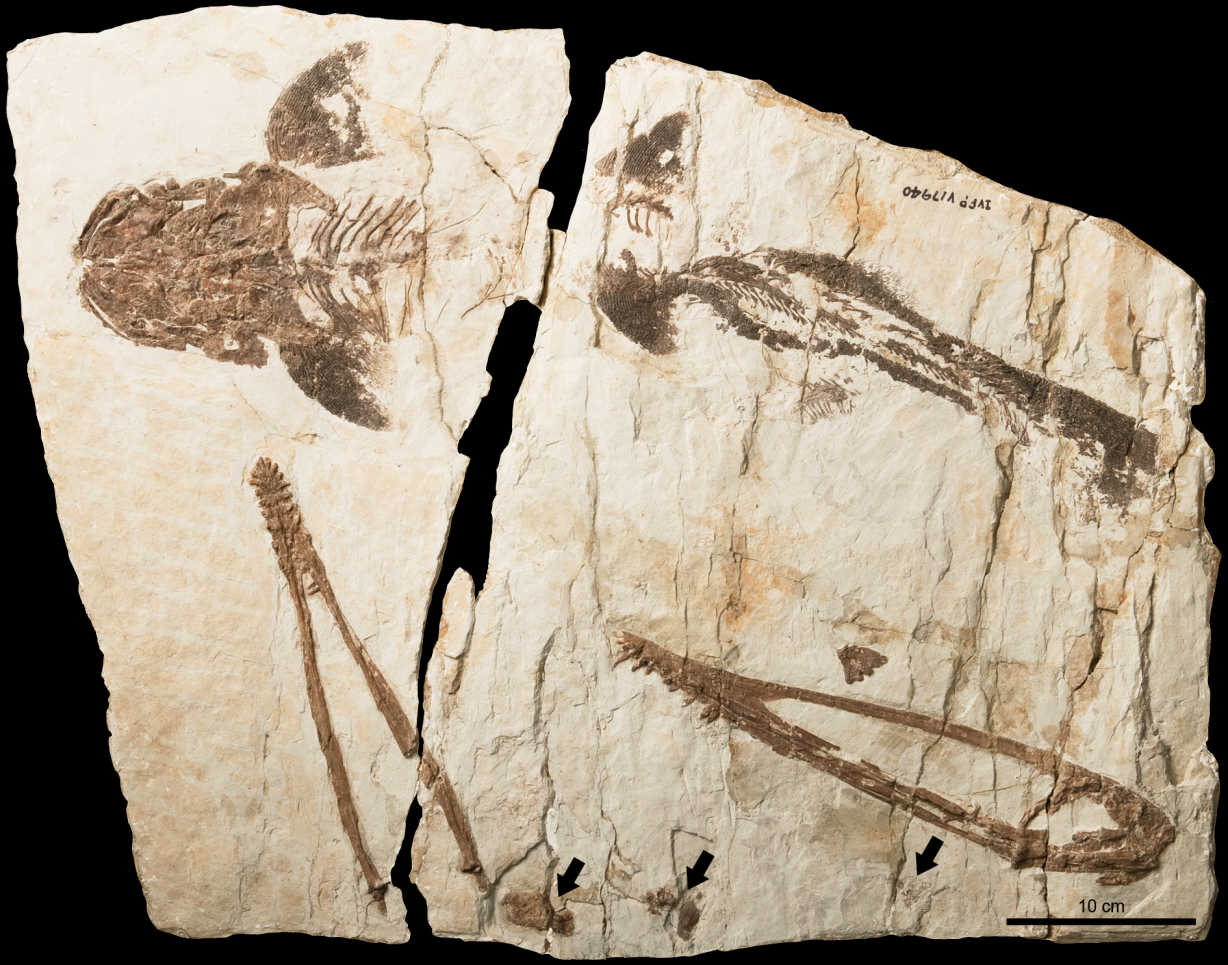
*Lingyuanopterus camposi* gen. et sp. nov., photograph of holotype IVPP V 17940. The arrows indicate the pellets which likely represent bromalites of *Lingyuanopterus*. Photo credit: Wei Gao.

**Table:** 

*Lingyuanopterus camposi* gen. et sp. nov.

**Etymology.** “*Lingyuan*”, referring the locality Lingyuan where the specimen was found; “*pterus*”, Greek, meaning “wing”; the specific name “*camposi* ” is dedicated to Brazilian vertebrate paleontologist Diogenes de Almeida Campos for his contribution to China-Brazil pterosaur collaborative research.

**Figure 2:**
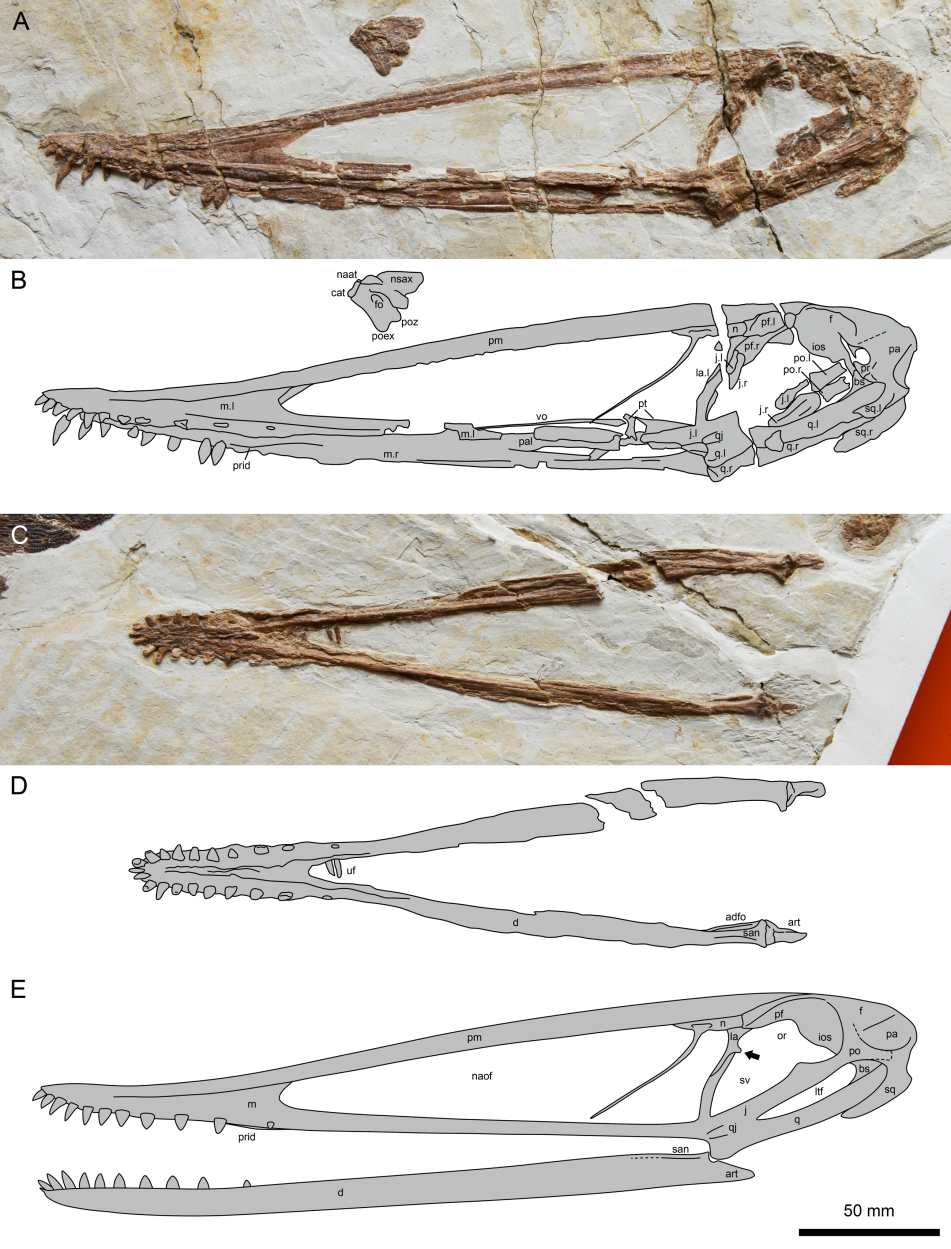
*Lingyuanopterus camposi* gen. et sp. nov., holotype IVPP V 17940. (A) Photograph of the skull and atlas-axis complex; (B) line drawing of the skull and atlas-axis complex; (C) photograph of the mandible; (D) line drawing of the mandible. (E) reconstruction of the skull and mandible of *Lingyuanopterus camposi*, position of the orbit process on the lacrimal indicated by the arrow is restored based on Micro-CL scans. Abbreviations: adfo, adductor fossa; art, articular; bs, basisphenoid; cat, centrum of atlas; d, dentary; f, frontal; fo, foramen; ios, interorbital septum; j, jugal; la, lacrimal; ltf, lower temporal fenestra; m, maxilla; n, nasal; naat, neural arch of atlas; naof, nasoantorbital fenestra; nsax, neural spine of axis; or, orbit; pa, parietal; pal, palatine; pf, prefrontal; pm, premaxilla; po, postorbital; poex, postexapophysis; poz, postzygapophysis; pr, prootic; prid, palatal ridge; pt, pterygoid; q, quadrate; qj, quadratojugal; san, surangular; sq, squamosal; sv, suborbital vacuity; uf, unknown fragments; vo, vomer; l, left; r, right. Photos credit: Xin Cheng.

**Figure 3:**
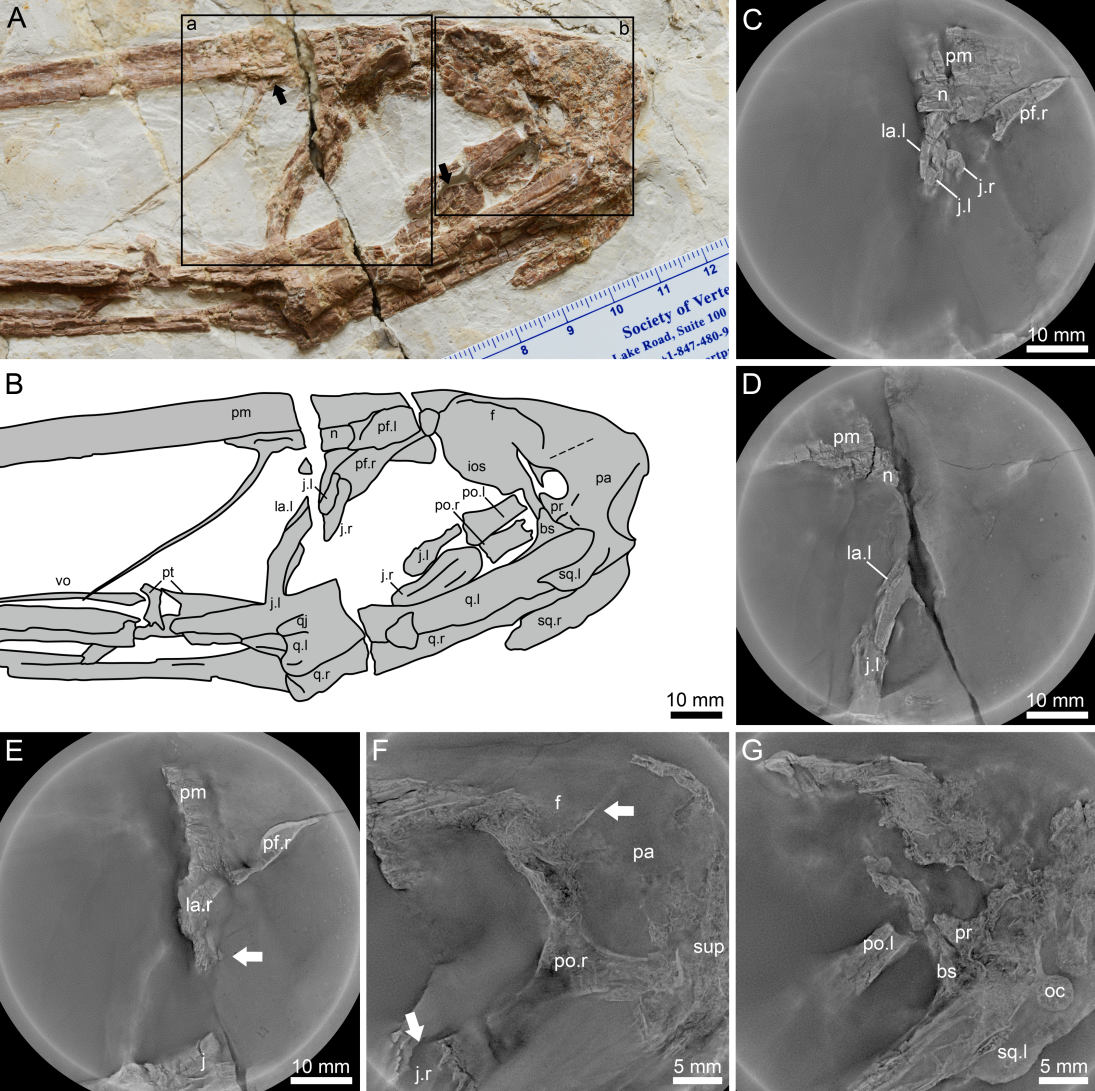
*Lingyuanopterus camposi* gen. et sp. nov., holotype IVPP V 17940, details of the posterior region of the skull. (A) Photograph of the posterior region of the skull, with the Micro-CL scanning images of frame a shown in C–E, and Micro-CL scanning images of frame b shown in F and G, the left arrow indicating the depression on the nasal, the right arrow indicating the flange on the anterodorsal margin of the postorbital process of the jugal; (B) line drawing of the bones in A; (C) Micro-CL scanning image, showing the contact condition of the premaxilla, nasal, lacrimal and the dorsal extension of the lacrimal process the jugal; (D) Micro-CL scanning image, showing the ventral extension of the jugal process of the lacrimal; (E) Micro-CL scanning image, showing a small bone fragment which represents the orbit process of the right lacrimal (bones on the right side of the skull are ventrally displaced); (F) Micro-CL scanning image, showing the right postorbital and surrounding bones, with the upper arrow indicating the boundary between the frontal and parietal, and the lower arrow indicating the flange on the anterodorsal margin of the postorbital process of the jugal; (G) Micro-CL scanning image, showing the prootic, dorsal end of the basisphenoid and surrounding bones. Abbreviations: bs, basisphenoid; f, frontal; ios, interorbital septum; j, jugal; la, lacrimal; m, maxilla; n, nasal; oc, occipital condyle; pa, parietal; pf, prefrontal; pm, premaxilla; po, postorbital; pr, prootic; pt, pterygoid; q, quadrate; sup, supraoccipital; sq, squamosal; vo, vomer ; l, left; r, right. Photos credit: Xin Cheng.

**Figure 4:**
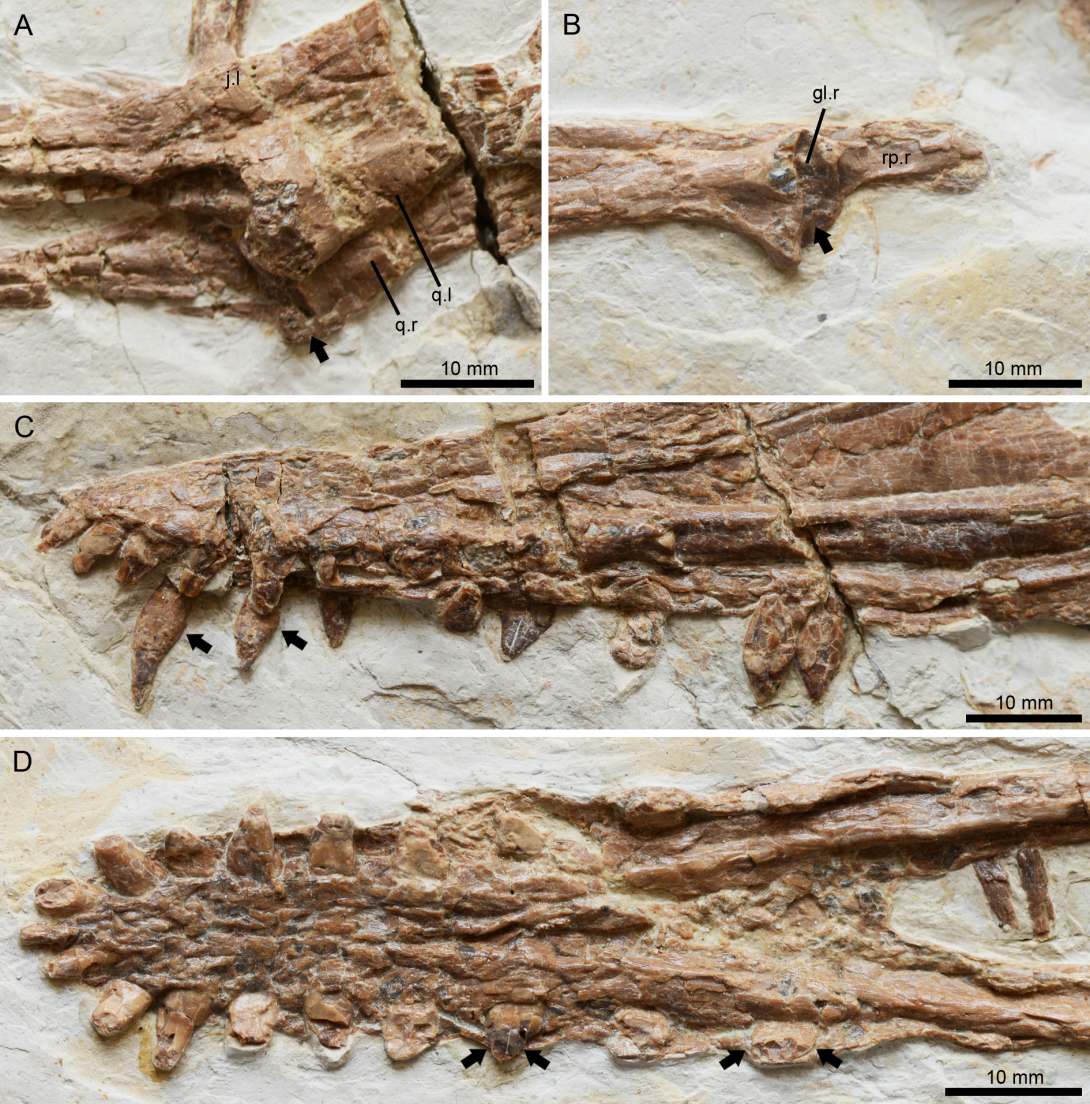
*Lingyuanopterus camposi* gen. et sp. nov., holotype IVPP V 17940, details of the helical jaw joint and teeth. (A) The slightly medially exposed right quadrate condyle divided by a groove (indicated by the arrow); (B) the right mandibular glenoid fossa divided by a weak ridge (indicated by the arrow); (C) the teeth of *Lingyuanopterus* on the upper jaw, with the arrows indicating the cingulum; (D) the teeth of *Lingyuanopterus* on the lower jaw, with the arrows indicating the mesial and distal carinae on the better preserved teeth. Abbreviations: gl, glenoid fossa; j, jugal; q, quadrate; rp, retroarticular process; l, left; r, right. Photos credit: Xin Cheng.

**Occurrence.** Sihedang, Lingyuan, Liaoning, China; Jiufotang Formation, Early Cretaceous (Aptian).

**Holotype.** IVPP V 17940 (Figs. 1–4), a near-complete skull, mandible and atlas-axis complex.

**Diagnosis.** Istiodactylid pterosaur that can be distinguished from others by the following characters (autapomorphies are marked with an asterisk): short triangular tooth crowns with sharp mesial and distal carinae limited to the distal teeth*; mandibular symphysis occupying approximately a quarter of the mandible length*; a flange on the anterodorsal margin of the postorbital process of the jugal separating the orbit and suborbital vacuity; the lacrimal process of the jugal slender and anterodorsally curved, extending more than 3/4 the height of the nasoantorbital fenestra; the jugal process of the lacrimal long and slender with an acute ventral end.

### Description

The holotype of *Lingyuanopterus* consists of a nearly complete skull exposed in the left lateral view (Figs. 2A–2B), a nearly complete mandible exposed in its dorsal view (Figs. 2C–2D), and an atlas-axis complex exposed in the left lateral view (Figs. 2A–2B). The skull is 306.8 mm long measured from the anterior tip of the premaxilla to the posterior margin of the squamosal, and 51.1 mm high measured from the highest point of the skull (above the anterior region of the frontal) to the jaw line. The nasoantorbital fenestra has a length of 149.3 mm at the ventral margin, occupying around 48.7% of the skull length. The dorsal margin of the skull is smooth and slightly curved, with no parietal crest/sagittal crest. The rostrum is slightly deflected dorsally, with its anterior end thicker at the ventral margin. The length of the skull anterior to the jaw articulation is 236.9 mm (see Table S1 for further measurements). The bones on the right side of the skull are ventrally displaced due to its preservation. The reconstruction of the skull is provided in Fig. 2E.

### Premaxilla and maxilla

The premaxilla and maxilla are extremely elongate, forming most of the skull length. The premaxilla constitutes most of the anterior and dorsal part of the skull and tapers to the anterior most tip, forming a pointed anterior end in lateral view. The posterior most extension of the premaxilla is unclear, but extends at least until the anterodorsal tip of the frontal in lateral view. The main body of the premaxilla is curved slightly dorsally, forming the dorsal margin of the skull. Most of the premaxilla-maxilla suture can be distinguished, except the anterior most part. Part of the posterior process of the maxilla is missing, and the preserved part is constant in height and slender compared to the premaxilla. In the region posteroventral to the rostrum, a narrow part of the palatal surface of the maxilla is exposed, forming a prominent median ridge more developed at the posterior part of the palate, as in other istiodactylids that preserve this part of the skull (Wang et al., 2005; Zhou et al., 2019; Averianov et al., 2021).

### Nasal and lacrimal

The nasal is positioned caudally above the nasoantorbital fenestra (Kellner & Tomida, 2000; Bennett, 2001). It dorsally contacts the premaxilla and is posteriorly covered by the prefrontal (Figs. 3A–3B). The nasal descending process is nearly completely preserved and extremely slender. It nearly extends to the ventral margin of the nasoantorbital fenestra and is anteriorly inclined, similar to that of *Zhenyuanopterus longiristris* and *Ikrandraco avatar* (Lü, 2010; Wang et al., 2014a). A small anteroposteriorly elongated depression is present on the nasal body (Fig. 3A).

The lacrimal dorsally contacts the nasal and is posterodorsally overlapped by the prefrontal. The nasal and the lacrimal have a straight contact margin, which is better observed under Micro-CL scanning (Fig. 3C). The jugal process of the lacrimal extends anteriorly along the lacrimal process of the jugal. It ventrally ends with an acute tip, and is approximately half the dorsoventral height of the nasoantorbital fenestra (Figs. 3A–3B, 3D). The dorsal part of the posterior margin of the left lacrimal is slightly abraded. A small bone fragment at the posterior margin of the jugal process of the right lacrimal was observed in Micro-CL scans, which represents the orbit process of the right lacrimal (Fig. 3E).

### Jugal

The jugal is a triradiate bone forming the maxillary process anteriorly, the lacrimal process dorsally and the postorbital process posterodorsally (Kellner & Tomida, 2000; Bennett, 2001). Both the maxillary process and the postorbital process of the left jugal are poorly preserved. The lacrimal process of the jugal is long and slender, extending more than 3/4 the dorsoventral height of the posterior margin of the nasoantorbital fenestra. The lacrimal process of the jugal is slightly anterodorsally curved with the ventral part subvertical, while the dorsal part is posteriorly inclined, making the posterior margin of the nasoantorbital fenestra curved and inclined in lateral view. The lacrimal processes of both the left and right jugals are exposed and have blunt dorsal processes, with the precise contact between it and adjacent elements observed using micro-CL scanning (Fig. 3C). Most of the postorbital process of the left jugal is missing. Only part of the postorbital process of the right jugal is visible and appears plate-shaped, with more details observed under micro-CL scanning (Fig. 3F). The postorbital process of the jugal has a flange on its anterodorsal margin (Figs. 3A and 3F), which together with the orbit process on the lacrimal dividing the anterior vacuity into an upper part (orbit) and a lower part (suborbital vacuity). The posterior margin of the postorbital process of the jugal is straight and smooth.

### Prefrontal, frontal and parietal

The left prefrontal and a small part of the right are exposed. The prefrontal forms the anterodorsal margin of the orbit. It overlaps the premaxilla, nasal and lacrimal posterolaterally, extending laterally to form a distinct lateral ridge. The posterior part of the prefrontal is crushed and the nature of this contact with the frontal is unclear.

The frontal forms most of the dorsal margin of the orbit, with a lateral ridge extending along its preserved length. The frontal connects the interorbital septum on the ventral side, while the boundary between the frontal and interorbital septum could not be observed. The suture between the frontal and parietal is difficult to discern on the surface, but CL scans indicate a straight posteriorly inclined boundary, anterior to the base of the jugal process of the postorbital (Fig. 3F). The dorsal margin of the parietal is curved and posteroventrally inclined, making the posterodorsal margin of the skull curved, and the supraoccipital lower, relative to the dorsoventrally highest point of the skull.

### Postorbital

The left postorbital is broken with only the jugal process preserved, while the postorbital on the right side is covered by the prootic, basisphenoid and parietal, but can be observed in CL scans (Fig. 3F). The postorbital is triradiate, with a dorsoventral frontal process dorsally, an anteroposterior squamosal process posteriorly, and an inclined jugal process anteroventrally. The jugal process is robust, and broader at its anteroventral end, making the anteroventral end more than twice as broad as the midsection. The squamosal process extends near the posterior margin of the upper temporal fenestra and ends with a dorsoventral margin.

### Squamosal

The squamosals on both sides are exposed (Figs. 3A–3B), and overlie the quadrates anteroventrally. The anterior process of the left squamosal is crushed and its contact with the postorbital is obscured. The otic process of the left squamosal is broken, but based on the right squamosal, the otic process has a blunt end, extending around 40% the length of the quadrate.

### Prootic and basisphenoid

The prootic was rarely described in pterosaurs, as this element was typically fused with surrounding bones and its shape was difficult to identify (Kellner, 1996; Codorniú, Paulina-Carabajal & Gianechini, 2015; Codorniú et al., 2016). In *Lingyuanopterus* the boundary of the prootic and surrounding bones is still partly visible. The prootic has an anterodorsal-posteroventrally elongated oval body and an ascending process extending from the posterior part (Figs. 3A–3B and 3G). It contacts the parietal dorsally and basisphenoid ventrally with the sutures partly visible, and does not contact the quadrate, as suggested in previous research (Kellner, 1996). In pterosaurs, the laterosphenoid has been suggested to contact the prootic posteriorly (Kellner, 1996), while in *Lingyuanopterus* the laterosphenoid is either missing or completely fused with the frontal, and thus could not be observed. Bordered by the frontal, parietal, prootic and interorbital septum there is an oval-shaped hole, where the dorsal and anterior border seems broken. Due to the ventral displacement of the bones on the right side, whether the hole penetrates the left and right sides of the cranium is unclear, even with CL scans. The formation of this hole is unusual, but probably the result of the missing laterosphenoid.

Most of the basisphenoid is overlapped by the quadrate, with only the anterodorsal tip exposed, which contacts the prootic dorsally (Fig. 3G). The basisphenoid has an anterodorsal process at the dorsal end, as observed in *Tapejara wellnhoferi* and *Rhamphorhynchus muensteri* (Kellner, 1996; Bonde & Leal, 2015).

### Quadrate and quadratojugal

The quadrates of both sides are exposed. The quadrate is long and robust, at least 67 mm in length. The quadrate is inclined at 155° with respect to the ventral margin of the skull, similar to other istiodactylids (*e.g.*, 150° in *Istiodactylus sinensis* and *N. luei*, 160° in *N. ignaciobritoi*, Andres & Ji, 2006; Zhou et al., 2019). The articular condyle is slightly enlarged, and ventrally divided by a groove (Fig. 4A). Most of the quadratojugal is completely fused with the jugal and the quadrate, with only the basal most part the sutures visible.

### Palatal elements

Most of the palatal elements could not be observed due to its preservation. The vomer and pterygoid are partly exposed. The vomer is long and slender, which should separate the choanae. The anterior extension of the choanae is not clear but extends to at least the middle region of the nasoantorbital fenestra. The pterygoid is crushed with only a small part visible posterior to the vomer, while its lateral and posterior part is covered by the maxillary process of the jugal.

### Mandible

The mandible is nearly completely preserved and exposed on its dorsal surface, with only the posteromedial part of the left retroarticular process missing (Figs. 2C–2D). The mandible is 248.6 mm long measured from the anterior tip to the posterior end of the articular. The dentaries of opposite sides are firmly fused to each other at the mandibular symphysis. The mandibular symphysis is 64.8 mm in length, occupying around 26% of the mandible length; its length is approximately four times its width. The mandibular symphysis has a wide groove at the posterior region corresponding to the palatal ridge formed by the maxillae. On the anterior end of the mandible there is a distinct odontoid process, which is smaller relative to the adjacent teeth. The caudal part of the right mandible ramus is crushed and the boundaries between bones are difficult to distinguish, while on the caudal part of the left mandible ramus, the suture between the surangular and dentary could be identified. The surangular is a longitudinal bone anteroposteriorly extended along the posterodorsal surface of the mandible. However, its anterior extension could not be distinguished. On the left ramus, the adductor fossa is exposed. The left glenoid fossa is crushed and poorly preserved, while on the right glenoid fossa a weak posterolaterally extended ridge could be recognized (Fig. 4B), corresponding to the groove on the quadrate. This ridge divides the mandible glenoid fossa into two fossae, with the medial one distinctly smaller and slightly medially oriented. This groove-ridge system indicates the presence of the helical jaw joint. Posterior to the glenoid, the retroarticular process is elongated, occupying around 5% of the mandible length, as is typical of istiodactylids (Witton, 2012). On the dorsal surface of the retroarticular process, there is a longitudinal medial ridge.

Two fragments are preserved posterior to the mandibular symphysis, unlikely to be teeth based on their shape and texture. The origins of these fragments remain unclear.

### Dentition

There are 11 teeth on each side of the upper jaw and 10 teeth on each side of the lower jaw. The tooth crowns of all preserved teeth are triangular in lateral view and labiolingually compressed, a common condition in istiodactylids (Witton, 2013), with the degree of compression increasing distally (Figs. 4C–4D). On the upper jaw, the anterior end of the rostrum is slightly deflected dorsally, making the first three alveoli slightly higher than the jaw line. The mesial four pairs of upper teeth are anteriorly inclined with their curvature increasing mesially, and the mesial most pair anteriorly inclined at approximately 135° to the long axis of the skull. The mesial teeth are closely spaced, with the teeth on the rostral end almost touching each other resulting in well-interlocked jaws during occlusion. These intervals increase distally, as is typical in most istiodactylids (Andres & Ji, 2006; Zhou et al., 2019; Hone et al., 2020; Averianov et al., 2021), with the distance between the ninth and tenth alveoli more than twice the width of the tenth tooth in *Lingyuanopterus*. The mesial most 4 pairs of teeth are gently curved, with their crowns around six mm in height and twice the crown width. By contrast, the distal tooth crowns are around four mm in height and almost four mm in width. The fourth, fifth teeth on the right side and the tenth teeth on both sides of the upper jaw are dislodged from their alveoli and their roots are exposed. The roots of the fourth and fifth teeth are bulbous, with lingual cingula and were observed on their lingual surfaces (Fig. 4C). In the tenth tooth positions, the roots are almost the mirror images of the crowns, but slightly taller.

The tooth row on the dentary is similar to the upper jaw, with the mesial teeth comparatively taller and the mesial most teeth labiolingually compressed to a lesser degree. By contrast, the distal teeth are comparatively shorter, mesiodistally wider and strongly labiolingually compressed. The mesial teeth are labially inclined, while the distal teeth are nearly upright. It is worth mentioning that the mesial teeth lack sharp carinae on their mesial or distal margins, but these were observed on distal teeth. The last pair of alveoli on the lower jaw are small, around half the height of the second last tooth pair. Similar, a small last tooth pair is also seen in *Nurhachius* and *Luchibang* (Zhou et al., 2019; Hone et al., 2020).

Of all the well exposed teeth, the tooth crowns are smooth, with no medial carina on the labial surface. The teeth also lack mesial and distal constrictions between the crowns and roots, a condition observed in *Nurhachius* (Wang et al., 2005; Zhou et al., 2019).

### Atlas-axis complex

The atlas-axis complex is the only preserved post-cranium element of *Lingyuanopterus*, has a length of 21 mm and a height of 25 mm (Figs. 2A–2B). The atlas is a small structure, comprising the atlantal intercentrum and the atlantal neural arch. The axial neural spine is triangular-shaped and tall. A pneumatic foramen is developed on the lateral surface of the axis centrum. The postexapophyses are distinct from the axis centrum. The suture between the atlas and axis is visible.

### Phylogenetic analysis

**Figure 5:**
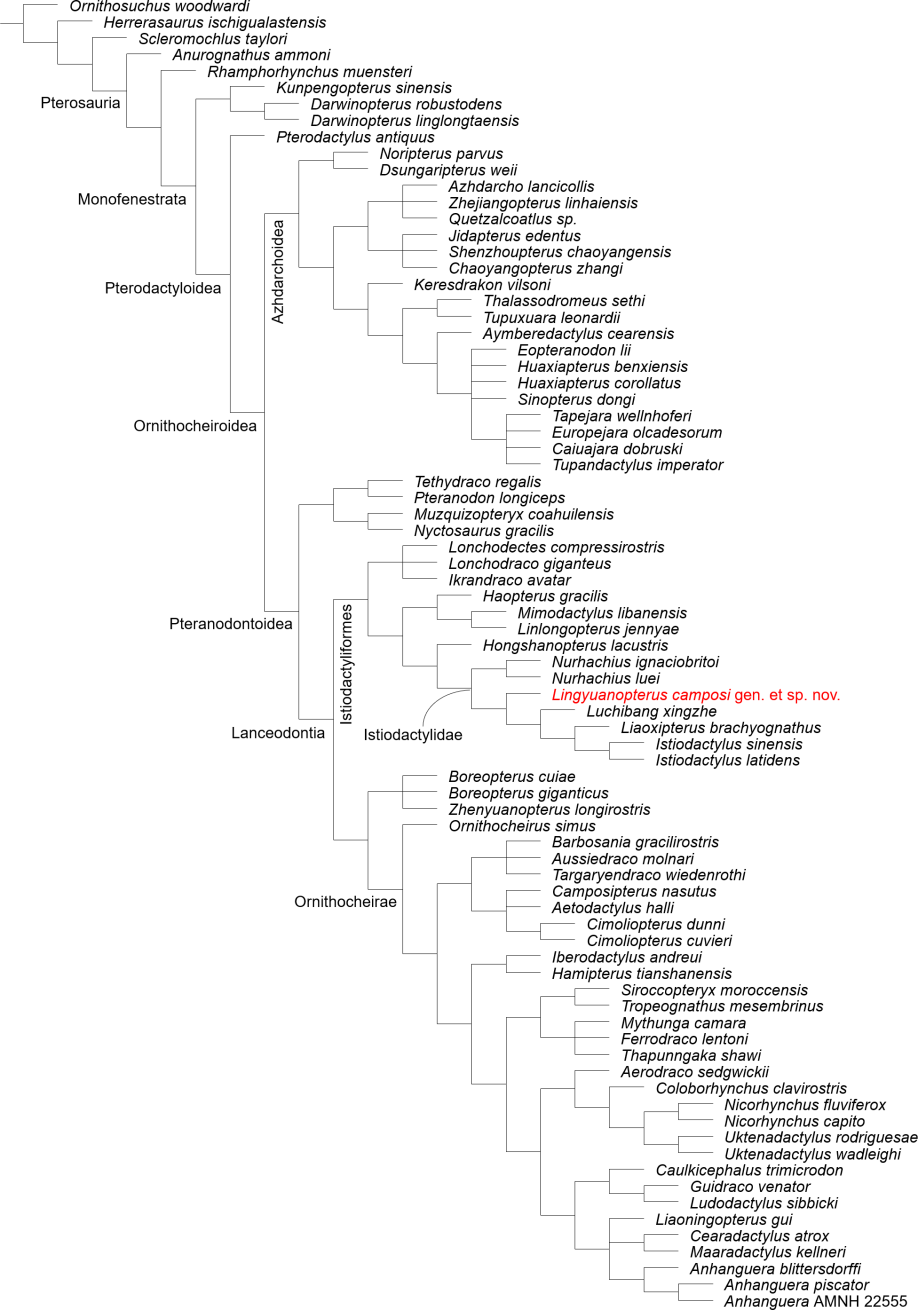
Phylogenetic relationships of *Lingyuanopterus camposi* gen. et sp. nov. Strict consensus tree of nine most parsimonious trees.

Our phylogenetic analysis produced 9 most parsimonious trees, with a minimum length of 437 steps, a consistency index of 0.596 and retention index of 0.857 (Fig. 5; see Fig. S1 for branch support values). *Lingyuanopterus* is resolved as the sister-taxon of *Luchibang* + (*Liaoxipterus* + *Istiodactylus*), together forming the clade Istiodactylinae (the most inclusive clade containing *Istiodactylus latidens* but not *N. ignaciobritoi*; Andres, Clark & Xu, 2014). They share the following synapomorphies: ventral margin of the orbit opened (char. 7), jugal posterior process orbital process present (char. 58), mandibular anterior end extension of the contact surface of opposing dentaries extended posteriorly less than 30% of mandible length (char. 91).

Andres, Clark & Xu (2014) proposed the branch-based definition of the Istiodactylidae as the least inclusive clade containing *Istiodactylus latidens* and *N. ignaciobritoi*. In our phylogenetic analysis, the clade Istiodactylidae share one synapomorphy among the features in the character list: palatal ridge more developed posteriorly (char. 74). Although only istiodactylids possess this character among istiodactyliforms, a palatal ridge becoming more pronounced posteriorly could also be seen in *Tropeognathus mesembrinus* (Wellnhofer, 1987) and *Ferrodraco lentoni* (Pentland et al., 2022) among pteranodontoids.

Kellner et al. (2019) proposed the clade Istiodactyliformes, containing istiodactylids and some associated taxa, and was defined as the most inclusive clade containing *Istiodactylus latidens*, but not *Anhanguera blittersdorffi*. Lonchodectids *Lonchodectes compressirostris*, *Ikrandraco avatar* and *Lonchodraco giganteus* are members of the Istiodactyliformes in our result, as supported by previous research (Zhou et al., 2019; Holgado & Pêgas, 2020; Richards, Stumkat & Salisbury, 2021). This gives the Istiodactyliformes a third family, Lonchodectidae (here we follow Rigal, Martill & Sweetman, 2017; Averianov, 2020 to use the term Lonchodectidae instead of Lonchodraconidae), along with the Istiodactylidae and Mimodactylidae (Kellner et al., 2019). Although most lonchodectids are represented only by fragmentary specimens, based on partial cranial materials (Averianov, 2020), the more complete skull of *Ikrandraco avatar* also has progressively laterally compressed teeth posteriorly on the tooth row. Moreover, the tooth crowns of *Lonchodraco giganteus* on the preserved rostrum and *Ikrandraco avatar* are relatively small with a size comparable to that of istiodactylids (Rodrigues & Kellner, 2013; Wang et al., 2014a; Martill et al., 2021). The tooth morphology of *Lonchodectes compressirostris* is unknown, but should also show a certain degree of lateral compression, based on the morphology of its alveoli (see Averianov, 2020, Fig. 1). In our analysis, the Istiodactyliformes has four synapomorphies: parietal ossified crest absent (char. 66), dentary tip odontoid process present (char. 98), teeth lateral compression present (char. 121), teeth cingulum present (char. 128). An odontoid process at the mandibular tip has also been reported in *Hamipterus tianshanensis* and *Targaryendraco wiedenrothi* (Wang et al., 2014b; Pêgas, Holgado & Leal, 2019).

## Discussion

### Comparison

*Lingyuanopterus* is a pterodactyloid based on the presence of a confluent naris and antorbital fenestra (Kellner, 2003). Although this feature is also seen in wukongopterids, *Lingyuanopterus* is distinguished from them by having a larger size and unique strongly labiolingually compressed teeth (Witton, 2013). *Lingyuanopterus* is further assigned to the Istiodactyliformes, based on the following features: teeth confined to the anterior half of the jaws, and labiolingually compressed crowns with a cingulum (Kellner et al., 2019).

The previous diagnoses of the Istiodactylidae were proposed only based on the description of *Istiodactylus*: the diagnosis Howse, Milner & Martill (2001) proposed was the same as the diagnosis of *Istiodactylus latidens*. The diagnosis Andres & Ji (2006) suggested includes the following features: nasoantorbital fenestra constituting over 58 percent of skull length and height, labiolingually compressed lancet-shaped teeth with truncated triangular roots. However, only in *Istiodactylus* the length ratio of nasoantorbital fenestra and skull reaches 58% (Wang et al., 2006; Andres & Ji, 2006; Witton, 2012; Zhou et al., 2019). Additionally, the teeth of *Nurhachius*, *Luchibang* and the mesial teeth of *Lingyuanopterus* are relatively labiolingually compressed to a smaller degree and lack the sharp mesial and distal carinae (Wang et al., 2005; Zhou et al., 2019; Hone et al., 2020). Thus, it is inappropriate refer to these as “lancet-shaped”. Moreover, some isolated teeth of *N. ignaciobritoi* have long and pointed tooth roots (Wang et al., 2005). Based on our phylogenetic analysis and the review of all reported istiodactylids, here we revise the diagnosis of the Istiodactylidae into the following features: istiodactyliforms with palatal ridge more developed posteriorly on the palate (synapomorphy), a large nasoantorbital fenestra occupying more than 50% of the length of the skull anterior to the jaw articulation, teeth strongly labiolingually compressed and restricted to the anterior part of the skull. *Lingyuanopterus* could be further assigned to the Istiodactylidae based on possessing a palatal ridge more developed posteriorly on the palate, a large nasoantorbital fenestra occupying 63% of the length of the skull anterior to the jaw articulation, and strongly labiolingually compressed teeth (although the mesial most few pairs of teeth are not strongly compressed) restricted to the anterior part of the skull. The holotype of *Lingyuanopterus* has a suture between the prootic and surrounding bones still partly visible, but its dentaries are fully fused together indicating this individual had at least reached the third ontogenetic stage according to the scheme proposed by Kellner (2015). Based on these evidences, the holotype is a subadult individual.

The nasoantorbital fenestra of *Lingyuanopterus* occupies 63% of the skull length anterior to the jaw articulation. Among the istiodactylids with this region completely preserved, this is a smaller ratio compared to *Istiodactylus* (around 68% in *Istiodactylus sinensis* and 73% in *Istiodactylus latidens*, Andres & Ji, 2006; Witton, 2012) but larger than *N. luei* (56%, Zhou et al., 2019), although these ratios could be affected by the ontogenetic variation of these specimens. *Lingyuanopterus* also has a rostral index (the length/height ratio of the rostrum anterior to the nasoantorbital fenestra, Martill & Naish, 2006) of 3.79, between *Istiodactylus* (approximately 2.9 in both species, Andres & Ji, 2006; Witton, 2012) and *Nurhachius* (4.2 in *N. ignaciobritoi* and 4.3 in *N. luei*, Wang et al., 2005; Zhou et al., 2019).

Of the istiodactylids from the Jehol Biota, only *Luchibang* was reported from the Yixian Formation. The holotype and only known specimen of *Luchibang* has the very tip of the snout and the posterior part of the skull missing (Hone et al., 2020). Therefore, only a few features of the skull could be compared with *Lingyuanopterus*. Although differing in age, the preserved part of the skull of *Luchibang* and *Lingyuanopterus* are quite similar. Both *Lingyuanopterus* and *Luchibang* have a low rostrum, slender mandibular ramus, and mandibular symphysis with a similar length-width ratio (Hone et al., 2020). However, *Lingyuanopterus* has a smooth and slightly curved skull dorsal margin with no crest developed, while in *Luchibang* there is a cranial crest on the posterior end of the preserved part (Hone et al., 2020). The teeth of *Luchibang* were described to be more robust than in other istiodactylids (Hone et al., 2020), similar to the mesial teeth in *Lingyuanopterus*, with both mesiodistally wider distally. However, on the teeth of *Luchibang* there are medial carinae on the labial surfaces (see Fig. S1 of Hone et al., 2020), which could not be seen in *Lingyuanopterus*. *Lingyuanopterus* is also different from *Luchibang* in that the mandibular symphysis of *Lingyuanopterus* is relatively longer (occupying 26% of the mandible length compared to 20% in *Luchibang* measured from the photo), and *Lingyuanopterus* has the anterior part of the jaw slightly tapered in occlusal view instead of being subparallel in *Luchibang* (Hone et al., 2020).

Records of *Istiodactylus* have been reported from the Vectis Formation of Britain (Howse, Milner & Martill, 2001) and Jiufotang Formation of China (Andres & Ji, 2006), with teeth assigned to this genus also reported from the Wessex Formation of Britain (Sweetman & Martill, 2010). *Lingyuanopterus* is similar to *Istiodactylus* in having a similar skull morphology; specifically, in terms of the morphology of the suborbital vacuity and the slender and posteriorly inclined jugal-lacrimal bar (Andres & Ji, 2006; Witton, 2012). However, the new taxon is distinguished from *Istiodactylus* in having a smaller tooth number (42 compared to 48 in *Istiodactylus latidens* and 60 in *Istiodactylus sinensis*; Howse, Milner & Martill, 2001; Andres & Ji, 2006), and the presence of a helical jaw joint (Howse, Milner & Martill, 2001; Andres & Ji, 2006). Moreover, the angle between the lacrimal and postorbital processes of the jugal are greater in *Lingyuanopterus*, making the suborbital vacuity broader than that of *Istiodactylus* (Andres & Ji, 2006; Witton, 2012). *Lingyuanopterus* differs further from *Istiodactylus sinensis* in having a pointed rostrum tip in lateral view, while in *Istiodactylus sinensis* the rostrum tip is blunter (Andres & Ji, 2006). The mandibular symphysis of *Lingyuanopterus* is slightly tapered in occlusal view, and also differs from *Istiodactylus latidens*, which comes to its widest at the fourth and fifth pairs of teeth, is slightly laterally depressed posteriorly and has a rounded rostral end (Averianov et al., 2021).

**Figure 6:**
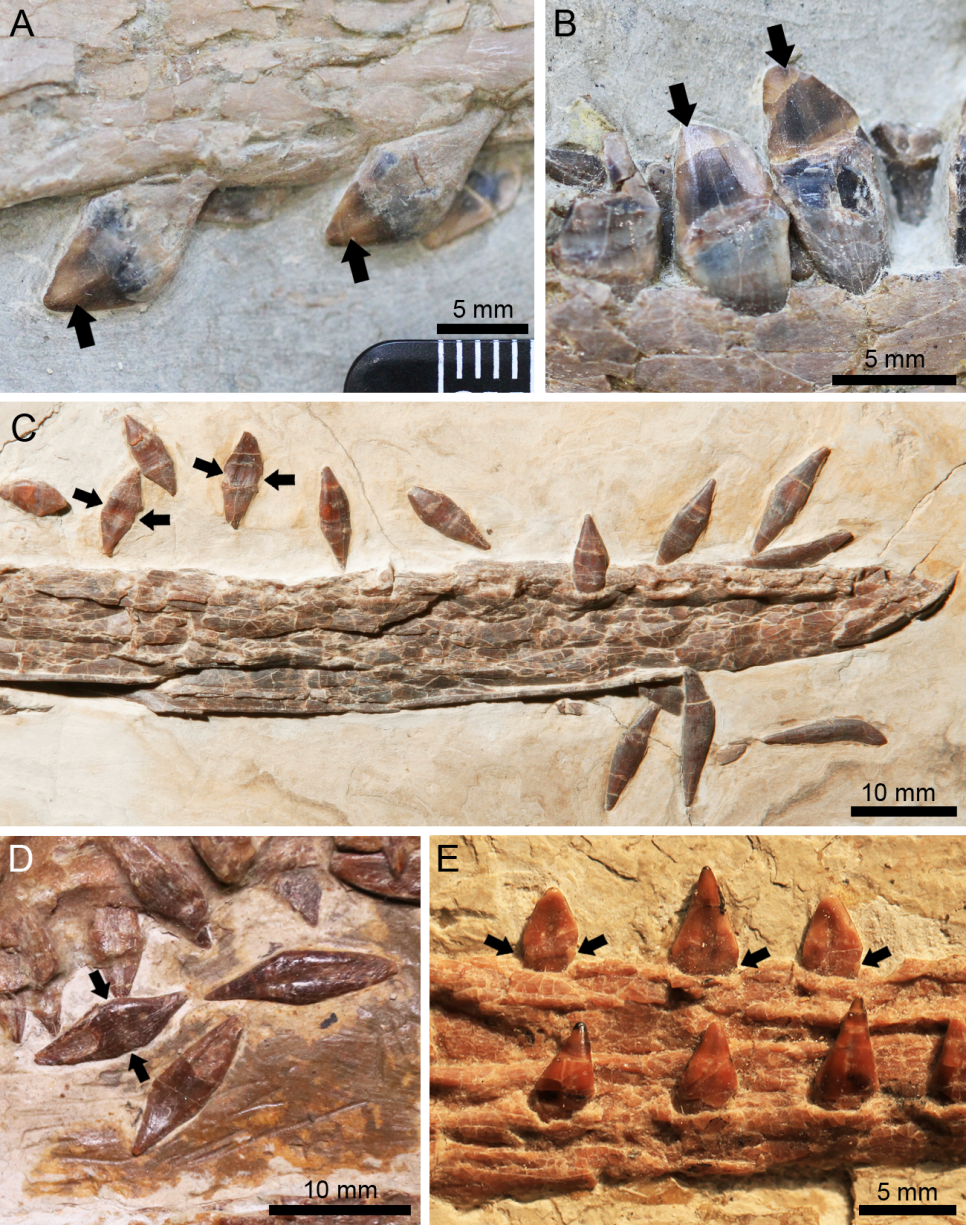
Different tooth forms of istiodactylids from the Jiufotang Formation. (A) The distal teeth of *Istiodactylus sinensis* GMC V2329 on the upper jaw. (B) The mesial teeth of *Istiodactylus sinensis* GMC V2329 on the lower jaw; (C) the teeth of *N. ignaciobritoi* IVPP V 13288 on the lower jaw; (D) the isolated teeth of *N. ignaciobritoi* PMOL-AP00003; (E) the distal teeth of *N. luei* BPMC-0204 on the lower jaw, modified from Zhou et al. (2019). The arrows in A and B indicate the labial medial carinae, the arrows in C-E indicate the mesiodistal constrictions between the crowns and roots.

The tooth morphology of *Lingyuanopterus* is also different from that of *Istiodactylus*. The teeth of *Istiodactylus* have medial carinae on the labial surfaces of their tooth crowns, which are formed by the surfaces between two slightly differently oriented planes of the crown labial surfaces (Figs. 6A–6B; Averianov et al., 2021). By contrast, the labial surfaces of the exposed crowns of *Lingyuanopterus* are smooth. *Lingyuanopterus* also has its anterior most few pairs of teeth on the lower jaw labiolingually compressed to a lesser degree compared to *Istiodactylus* (Andres & Ji, 2006; Averianov et al., 2021). Moreover, in *Lingyuanopterus*, *Luchibang* and *Nurhachius* the teeth show a distinct pattern in which the crowns become mesial-distally narrower to the anterior end of both upper and lower jaw. However, in *Istiodactylus* this tendency, if present, is not obvious.

Another istiodactylid from the Jiufotang Formation, *Liaoxipterus*, is only know from an incompletely preserved lower jaw, exposed on the dorsal view (Dong & Lü, 2005; Lü, Xu & Ji, 2008). Compared with *Liaoxipterus*, the mandibular symphysis of *Lingyuanopterus* is distinctly narrower, being approximately 4 times as long as its width, while in *Liaoxipterus* the ratio is around 2.8 (Hone et al., 2020). *Lingyuanopterus* also has a smaller number of teeth (20 compared to 26 on the lower jaw), with only the last pair of teeth posterior to the posterior end of the mandibular symphysis, instead of three pairs in *Liaoxipterus* (Lü, Xu & Ji, 2008). The mandibular symphysis of *Lingyuanopterus* is slightly tapered, while *Liaoxipterus* has a subparallel mandibular symphysis margin with a rounded rostral end, as in *Istiodactylus* (Witton, 2012).

A close relationship has previously been suggested between *Liaoxipterus* and *Istiodactylus sinensis* (Witton, 2012). By contrast, Lü, Xu & Ji (2008) stated that *Liaoxipterus* differs from *Istiodactylus* in the absence of medial carinae on the tooth crowns in the former. However, the medial carinae of *Istiodactylus* are on the labial surfaces (Andres & Ji, 2006; Averianov et al., 2021). Therefore, it could not be observed in *Liaoxipterus*, since the only specimen has its teeth only exposed on the lingual view, so the presence of medial carinae cannot be confirmed. The differences between *Liaoxipterus* and both species of *Istiodactylus* are quite limited. The interrelationship between these two taxa warrants further study. but is beyond the scope of the present study.

*Nurhachius* is another genus of istiodactylids from the Jiufotang Formation, with some contention surrounding its taxonomic validity. It has been suggested that *Longchengpterus zhaoi* (PMOL-AP00003; Wang et al., 2006), is a junior synonym of *N. ignaciobritoi* for having identical skull shape and tooth morphology (Lü, Xu & Ji, 2008) and sharing several features of the skull (Zhou et al., 2019). These assertions have been rejected by Witton (2012) and Hone et al. (2020). Witton (2012) proposed that *Longchengpterus zhaoi* differs from *N. ignaciobritoi* in having smaller interalveolar spaces (in *N. ignaciobritoi* the majority of the interalveolar spaces are greater than the width of the adjacent tooth), fewer teeth and a relatively shorter tooth row in his phylogenetic analysis. However, the interalveolar spaces of all well exposed alveoli are closely arranged in *N. ignaciobritoi*. While the exact tooth number of *Longchengpterus zhaoi* remains uncertain, 12 teeth were observed on the right dentary in the original diagnosis of that taxon (Wang et al., 2006); Lü, Xu & Ji (2008) observed 9 tooth pairs on the lower jaw, whereas 12 pairs of teeth on the upper jaw were observed by Zhou et al. (2019). Based on our observation of specimen PMOL-AP00003, the exact tooth number on the upper jaw could not be distinguished because of crushing, and there are 11 pairs of teeth on the lower jaw. It has also been suggested by Hone et al. (2020) that *N. ignaciobritoi* has a proportionally smaller skull, as it has a smaller skull-humerus length proportion (2.6) compared to *Longchengpterus zhaoi* (3.3). Although the holotype of *N. ignaciobritoi* actually has the posterior part of its skull missing and the total length of the skull is unknown, the length ratio of the length of the skull anterior to the jaw articulation to the humerus is 2.3 in *N. ignaciobritoi* compared to 2.4 in *Longchengpterus zhaoi*. Thus, the difference in skull/humerus length proportion is indistinct. Notably, both *Nurhachius* and *Longchengpterus zhaoi* have a distinct tooth morphology, such that the teeth are slightly constricted mesiodistally between the crowns and roots, and the crowns have shallow concavities on the basal part of the crowns (Figs. 6C–6E; Wang et al., 2005; Wang et al., 2006; Zhou et al., 2019). We follow the previous opinion of Lü, Xu & Ji (2008) and Zhou et al. (2019) that “*Longchengpterus zhaoi*” is a junior synonym of *N. ignaciobritoi*, making this species represented by two specimens, the holotype (IVPP V 13288) and referred specimen, PMOL-AP00003.

Zhou et al. (2019) reported another species of *Nurhachius*, *N. luei*. However, it was suggested by Hone et al. (2020) that *N. luei* actually has a phylogenetic position outside the Istiodactylidae, instead of being a species of *Nurhachius*. This is primarily based on its craniomandibular articular positioned near the center of the orbit and its tooth row extending more than a quarter of the jaw length (Hone et al., 2020). Based on our phylogenetic analysis, *N. luei*, is still resolved within the Istiodactylidae as a sister taxon of *N. ignaciobritoi*. In the only specimen of *N. luei* the skull is fractured with the posterodorsal part slightly anterodorsally dislodged, the craniomandibular articular is actually positioned below the anterior end of the orbit after restoring this dislocation. *N. luei* has a distinct tooth morphology in that it also possesses mesiodistal constrictions between crowns and roots, and shallow concavities in the basal part on the crowns (Zhou et al., 2019). Thus, here we still maintain *N. luei* as a species of *Nurhachius*.

*Lingyuanopterus* is similar with both species of *Nurhachius* in the general skull morphology. However, both species of *Nurhachius* lack the suborbital vacuity, and have relatively short lacrimal process of the jugal, extending slightly more than half the height of the nasoantorbital fenestra (Wang et al., 2005; Zhou et al., 2019). By contrast, *Lingyuanopterus* has a long, curved and slender lacrimal process of the jugal extending more than 3/4 the height of the nasoantorbital fenestra. *Lingyuanopterus* also has fewer teeth and lacks the unique tooth morphology of *Nurhachius*. Moreover, the mandibular symphysis of *Lingyuanopterus* is relatively shorter, occupying 26% of the jaw length while in *N. ignaciobritoi* the ratio is 34% and in *N. luei* the ratio is 36% (Wang et al., 2005; Wang et al., 2006; Zhou et al., 2019).

Another pterosaur from the Jiufotang Formation, *Hongshanopterus*, was originally reported as a primitive istiodactylid (Wang et al., 2008). However, based on the branch-based definition of the Istiodactylidae proposed by Andres, Clark & Xu (2014) and its phylogenetic position in our result, *Hongshanopterus* is not a member of the Istiodactylidae, but a sister taxon of this clade, as suggested by previous research (Zhou et al., 2019; Kellner et al., 2019). The palatal ridge of *Hongshanopterus* is long and low, instead of being more developed posteriorly as in istiodactylids. The holotype and only known specimen of *Hongshanopterus* preserves the skull in palatal view. Hence, it has very little anatomical overlap with *Lingyuanopterus*. Although both have labiolingually compressed tooth crowns, *Lingyuanopterus* differs further from *Hongshanopterus* in having distinctly fewer teeth (22 compared to 34–38 on the upper jaw) with a distinctly shorter tooth row (Wang et al., 2008).

Although the number of istiodactylids from the Jehol Biota might still be controversial due to ongoing debates regarding the validity of some species (Lü, Xu & Ji, 2008; Witton, 2012; Zhou et al., 2019; Hone et al., 2020), our results and the discussions herein highlight this was a diverse clade. Istiodactylids from the Jiufotang Formation have shown at least three different types of teeth: strongly labiolingually compressed triangular crowns with sharp mesial and distal carinae (lancet-shaped) and medial carinae on the labial surfaces (Figs. 6A–6B, *Istiodactylus sinensis*), labiolingually compressed triangular crowns with sharp mesial and distal carinae restricted to the distal teeth (Figs. 4C–4D, a new type of tooth morphology only represented by *Lingyuanopterus*), and labiolingually compressed triangular crowns with slight mesiodistal constrictions between crowns and roots and shallow concavities on the basal part of the crowns (Figs. 6C–6E, *Nurhachius*). In both the latter two types, the tooth crowns also show a distinct tendency of becoming more labiolingually compressed and mesial-distally wider distally. The known features of the teeth of *Liaoxipterus* are quite limited, but are most similar to the first type based on the holotype specimen (Witton, 2012). Similar diverse tooth crown forms could also be seen in the isolated istiodactylid teeth from the Wessex Formation of Britain, Barremian (Sweetman & Martill, 2010).

### Helical jaw joint

**Figure 7:**
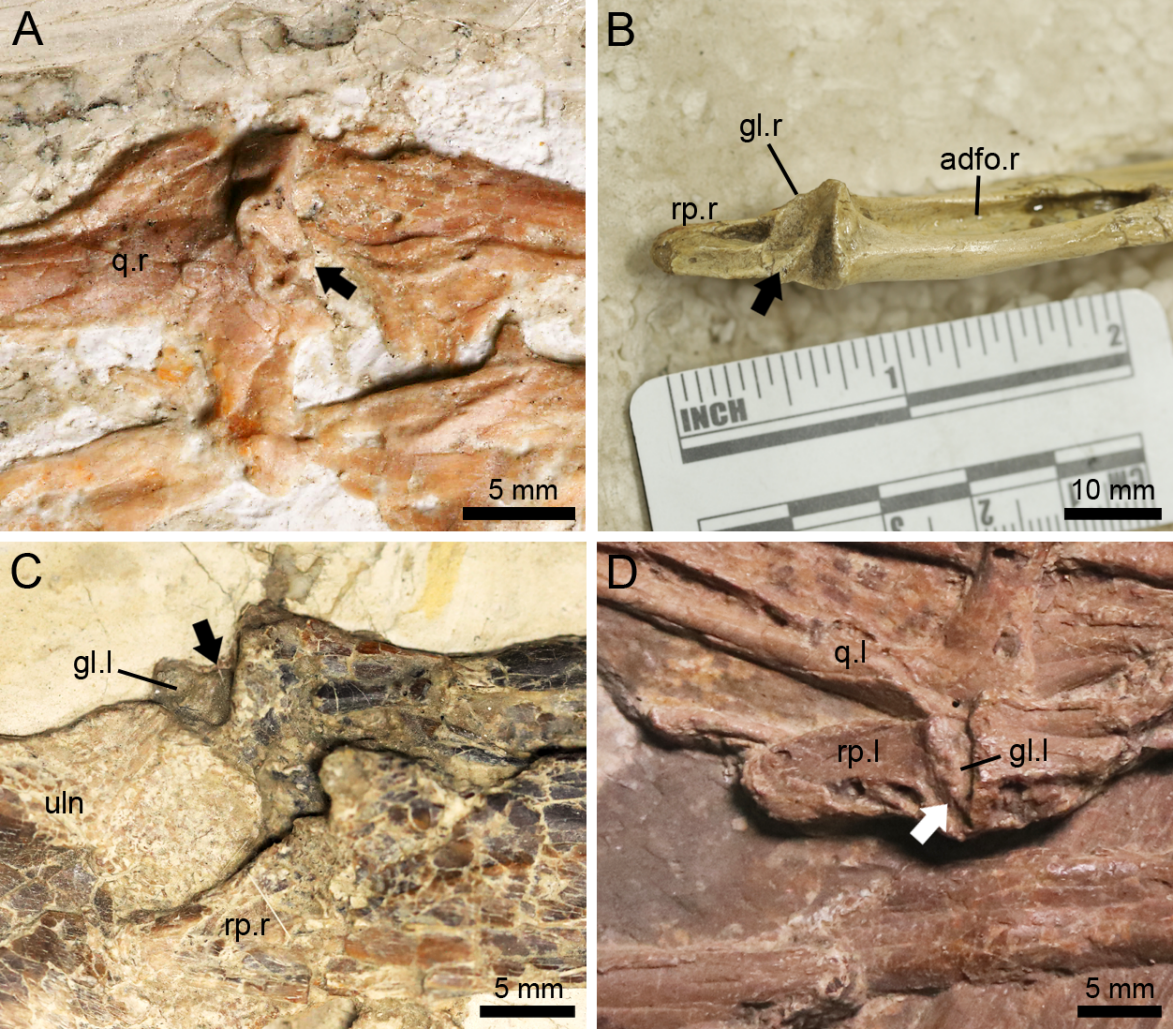
The helical jaw joints in pterosaurs. (A) Articular condyle on the right quadrate of *Hongshanopterus*, showing a typical helical jaw joint as a diagonal groove dividing the quadrate condyle; (B) The three-dimensionally preserved right mandibular glenoid fossa of *Hamipterus* IVPP V 18936.3, showing a typical helical jaw joint as a diagonal ridge dividing the mandibular glenoid fossa; (C) The structure of the helical jaw joint of *N. ignaciobritoi* IVPP V 13288 on the left mandibular glenoid fossa; (D) The structure of the helical jaw joint of *N. ignaciobritoi* PMOL-AP00003 on the left mandibular glenoid fossa. The arrows indicate the groove on the quadrate condyle or ridge on the mandibular glenoid fossa. Abbreviations: adfo, adductor fossa; gl, glenoid fossa; q, quadrate; rp, retroarticular process; uln, ulna; l, left; r, right.

The helical jaw joint is a feature present in many ornithocheiroids (Wellnhofer, 1985; Buffetaut, Ősi & Prondavi, 2011). This structure includes the subcylindrical condyle on the quadrate divided by a diagonal groove extending diagonally across it (Bennett, 2001), as seen in the sister taxon of the Istiodactylidae, *Hongshanopterus* (Fig. 7A). The concave glenoid fossa on the mandible is also divided by a corresponding ridge (Bennett, 2001), which can be seen in the three-dimensionally preserved *Hamipterus tianshanensis* (Fig. 7B).

The Istiodactylidae was previously described as a clade without helical jaw joint (Witton, 2012; Hone et al., 2020). However, based on our observation, the holotype of *N. ignaciobritoi* has its left jaw joint on the mandible slightly medially exposed (Fig. 7C), which shows a distinct ridge dividing the glenoid fossa into a medial and a lateral part, corresponding to the helical jaw joint described by previous researchers (Wellnhofer, 1980; Bennett, 2001). Moreover, a helical jaw joint is also present in the referred specimen of *N. ignaciobritoi* (PMOL-AP00003) based on our observation. Although the quadrate condyles of both sides are covered by the surrounding bones and the right mandibular glenoid fossa is not exposed due to its preservation, the well exposed left mandibular glenoid fossa also shows the distinct structure of helical jaw joint as a diagonally oriented ridge dividing the glenoid fossa into two parts (Fig. 7D).

*Lingyuanopterus* also shows a helical jaw joint, with a distinct groove dividing the convex articular condyle on the quadrate (Fig. 4A). A weak posterolaterally extended ridge could also be observed on the glenoid fossa of the better-preserved right mandible (Fig. 4B). The ridge only extends to the middle of the posterior margin of the glenoid fossa, and thus differs from the condition of *Quetzalcoatlus* sp., “*Ornithocheirus buenzeli*”, *Hamipterus tianshanensis* and *Nurhachius* where the long ridge extends across the whole glenoid fossa (Kellner & Langston Jr, 1996; Buffetaut, Ősi & Prondavi, 2011; Wang et al., 2014b).

The function of this structure has been discussed by several authors before. It has been suggested that the helical jaw joint could spread both rami of the mandible which allows the lower jaw to open wider and extend a throat sac, similar to the extant *Pelecanus* (Eaton, 1910). However, this assertion was rejected by Witton & Naish (2015) who suggest that the lateral extension is quite limited. This spread of both mandible rami was also suggested to allow the retroarticular process to pass the quadrate laterally and the jaw open at a greater angle (Wellnhofer, 1980). Mover, Fastnacht (2005) further illustrated that the helical jaw joint could store elastic energy during jaw opening process, since it slightly spreads the mandibular rami and releases the stored elastic energy during jaw closing process, causing a greater acceleration (Fastnacht, 2005). However, Bennett (2001) rejected the view that the helical jaw joint could spread the mandible, but suggested that this configuration could maintain accurate alignment at the jaw joints, since a small misalignment could lead to a big displacement at the tip of the jaw. This could also function as a resistance of the posteromedial pull of *M. adductor mandibulae* based on the study of *Pteranodon* (Bennett, 2001). The rostral end of the mandible in *Lingyuanopterus* and *Nurhachius* are relatively narrower and slightly tapered compared to more derived istiodactylids such as *Istiodactylus* (Witton, 2012; Zhou et al., 2019; Averianov et al., 2021). Therefore, these taxa might need a more constrained helical jaw joint to ensure a precise occlusive alignment at the anterior end of the rostrum.

### Ecology

There have been several hypotheses suggested on the diets of istiodactylids, including piscivory (Hooley, 1913; Wellnhofer, 1991; Wang & Zhou, 2006), herpetivory (Hooley, 1913), possible filter-feeding (Fastnacht, 2005) and insectivory (based on the lingual process of *Liaoxipterus*, Lü, 2015, although rejected by Jiang et al., 2020). However, more evidences have suggested a scavenging diet, based on several features including the well-interlocked labiolingually compressed teeth, slender maxillae, shallow rostra and mandibular symphyses, long retroarticular processes (Howse, Milner & Martill, 2001; Wang & Zhou, 2006; Witton, 2012; Martill, 2014). *Istiodactylus* possesses several adaptations suited for scavenging, including teeth with razor edges (sharp mesial and distal carinae), weak orbit-surrounding bones in both species, and the elongate, wide occipital face and deep skull in *Istiodactylus latidens* (Ősi, 2011; Witton, 2012; Martill, 2014). The scavenging hypothesis is also supported by the study of dental microwear texture analysis of *Istiodactylus latidens* (Bestwick et al., 2020). Although the non-lancet-shaped tooth crowns in *Nurhachius* and the mesial teeth of *Lingyuanopterus* seem less adapted for cutting meat from corpses as suggested in *Istiodactylus*, the overall differences in tooth morphology and other cranial elements among istiodactylids from the Jiufotang Formation are quite limited. Modern scavenging vultures coexisting with one another in a single habitat demonstrate different styles and differ from each other in terms of their skull morphologies and sizes (Hertel, 1994). A similar pattern might also occur in istiodactylids from the Jiufotang Formation.

**Figure 8:**
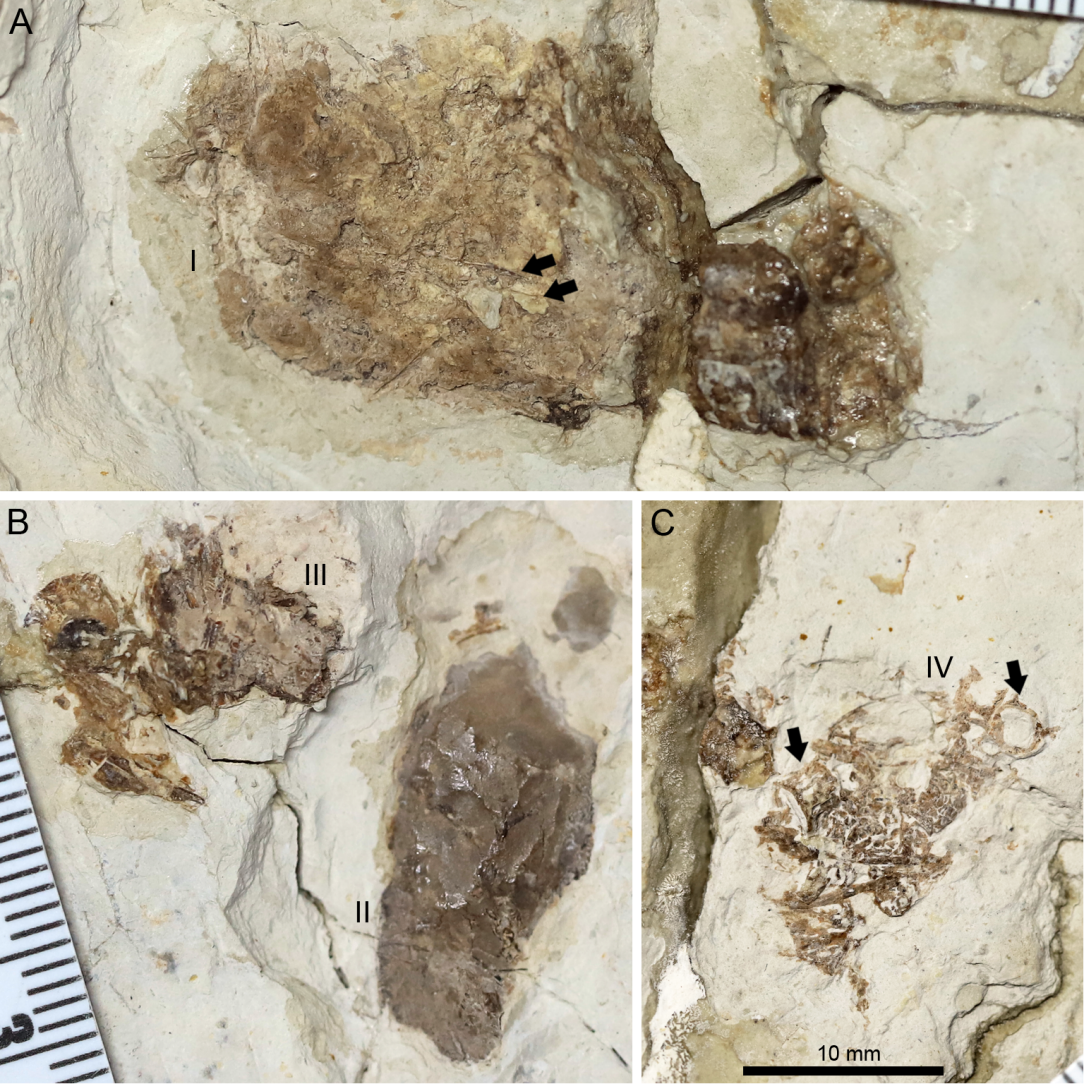
Photographs of the pellets tentatively interpreted as bromalites of *Lingyuanopterus camposi* gen. et sp. nov. (A) Photograph of pellet I, fragments of probable fish ribs are indicated by arrows; (B) photograph of pellets II and III; (C) photograph of pellet IV, fish vertebrae are indicated by arrows. Their positions on the fossil are pointed in Fig. 1. The three photographs are the same scale.

A peipiaosteid fish (Fig. 1; Grande & Bemis, 1996) and four pellets are preserved together with *Lingyuanopterus* (Fig. 8). Pellet I is four cm long and 1.9 cm wide, the largest among the four pellets, with two long and slender bone fragments, regarded here as fish ribs (Fig. 8A). Pellet II and III are close together and both dark brown colored (Fig. 8B). Pellet II has a smooth outline and contains few fragments, while pellet III contains many more fragments, although the taxonomic and morphologic affinities of this material cannot be stated with certainty. Pellet IV is different from other three pellets in containing clear fragments including fish vertebrae (Fig. 8C). Among fish in the Jiufotang Formation, acipenseriforms do not possess ossified centra as observed in pellet IV (Grande & Bemis, 1996). Although *Sinamia* possess ganoin scales (Yabumoto, 2017) which are easily preserved in fossils (similar fish scales have been seen in emetolites of *Kunpengopterus*, Jiang et al., 2022), these were not observed with *Lingyuanopterus*. The fish vertebrae could be assigned to the teleost fish, most probably *Jinanichthys* (Ma & Sun, 1988) since it matches the size of the vertebrae and are abundant in the Jiufotang Formation (Jin, 1996). Since the skull, lower jaw and post-cranium skeletons of *Lingyuanopterus* are separate, this preservation could be a taphonomic artifact. However, since these pellets are close together with the skull, these pellets are mostly likely bromalites (Hunt & Lucas, 2012) of *Lingyuanopterus*. Among different bromalite types, these pellets seem unlikely to be coprolites, since the fragments in pterosaur coprolites are usually quite small (Wellnhofer, 1975; Hone et al., 2015; Qvarnström et al., 2019). Although istiodactylids were mostly considered as scavengers, These putative bromalites could be a result of scavenging on fish corpses. Alternatively, since the mesial most few pairs of teeth in *Lingyuanopterus* show less adaption of cutting corpses than that in *Istiodactylus*, it is possible that *Lingyuanopterus* also show certain degree of piscivory.

Various morphotypes of istiodactylid teeth have also been reported in the Wessex Formation from Britain (Sweetman & Martill, 2010). Although this geologic formation is older than the Jiufotang Formation, the pterosaur fauna of the Wessex Formation is similar when compared with the former (Wang & Zhou, 2006), comprising the Ctenochasmatidae (Sweetman & Martill, 2010), Ornithocheiridae (Steel et al., 2005; Martill, 2015), Tapejaridae (Martill et al., 2020) and diverse istiodactylids (Sweetman & Martill, 2010). The Wessex Formation is interpreted as a meander-belt floodplain deposit, with a diverse fish and tetrapod fauna (Steel et al., 2005). These ecosystems may have been ideal for istiodactylids, and enabled members of this clade to develop different feeding strategies similar to the Jiufotang Formation.

## Conclusions

*Lingyuanopterus* is a new member of the clade Istiodactylidae, and represents the fifth species of istiodactylids from the Jiufotang Formation. We revise the diagnosis of the Istiodactylidae and for the first time, we record the presence of helical jaw joints in istiodactylids. The different types of tooth morphologies and the presence of helical jaw joints in some istiodactylids indicate members of this clade probably demonstrated diverse feeding behaviours. These different morphologies might be similar to the ecologies of modern scavenging birds which demonstrate diversity and various feeding styles in a single habitat. The presence of pellets containing fish fragments with the holotype are interpreted as probable bromalites of *Lingyuanopterus*, which may represent this pterosaur scavenging on fish corpses or showing a certain degree of piscivory. The diverse istiodactylids and other pterosaur records indicate a similarity in terms of composition at the family level between the pterosaur assemblages of the Jiufotang Formation (Aptian) from northeastern China and the earlier Wessex Formation (Barremian) from Britain.

## Supplemental Information

10.7717/peerj.13819/supp-1 Supplemental Information 1 Measurements of *Lingyuanopterus camposi* gen. et sp. nov., IVPP V 17940 and phylogenetic tree of ornithocheiroids with branch support values

10.7717/peerj.13819/supp-2 Supplemental Information 2 Character codings for the phylogenetic analysis of the Ornithocheiroidea

## Institutional abbreviations

BPMC: Beipiao Pterosaur Museum of China, Beipiao, Liaoning Province, China
IVPP: Institute of Vertebrate Paleontology and Paleoanthropology, Chinese Academy of Sciences, Beijing, China
GMC: the Geological Museum of China, Beijing, China
PMOL: Paleontological Museum of Liaoning, Shenyang, China

## Additional Information and Declarations

### Data Availability

The following information was supplied regarding data availability: The raw data is available in the Supplemental File.

### New Species Registration

The following information was supplied regarding the registration of a newly described species: Publication LSID: urn:lsid:zoobank.org:pub:57EAFE78-9764-4777-A9DF-328325419767. Lingyuanopterus genus name: urn:lsid:zoobank.org:act:9F380C64-50BA-4B1F-8885-468362E0BE46. Lingyuanopterus camposi species name: urn:lsid:zoobank.org:act:B5D251D4-B65C-48E2-A00F-08DB29D09732.
