# Supplementary material for: A new istiodactylid pterosaur, Lingyuanopterus camposi gen. et sp. nov., from the Jiufotang Formation of western Liaoning, China
Source: PeerJ. 2022 Jul 26;10:e13819. doi: 10.7717/peerj.13819 (PMC9336611; doi:10.7717/peerj.13819)

**A new istiodactylid pterosaur, *Lingyuanopterus*** ***camposi* gen. et sp. nov., from the Jiufotang Formation of western Liaoning, China**

Yizhi Xu ^1,2,3^, Shunxing Jiang ^1,3^, Xiaolin Wang ^1,2,3^

^1^ Key Laboratory of Vertebrate Evolution and Human Origins, Institute of Vertebrate Paleontology and Paleoanthropology, Chinese Academy of Sciences, Beijing, China

^2^ College of Earth and Planetary Sciences, University of Chinese Academy of Sciences, Beijing, China

^3^ CAS Center for Excellence in Life and Paleoenvironment, Beijing, China

**Supplementary Information**

**Table S1. Measurments of *Lingyuanopterus camposi* gen. et sp. nov., IVPP V 17940.**

| **Dimensions** | **Measurment (mm)** |
| --- | --- |
| Skull length | 306.8 |
| Mandible length | 248.6 |
| Pre-joint skull length^[1]^ | 236.9 |
| Skull dorsoventral height | 51.1 |
| Mandibular symphysis length | 64.8 |
| Rostral length | 83.5 |
| Anteroposterior length of the nasoantorbital fenestra (ventral margin) | 149.3 |
| Dorsoventral height of nasoantorbital fenestra | 35.6 |
| Upper tooth row length^[2]^ | 83.8 |
| Lower tooth row length | 71.9 |

[1] The length measured from the skull rostral tip to the anterior end of the jaw articulation.

[2] measured from the anterior margin of the first alveoli to the posterior margin of the last alveoli.

**Figure S1. Phylogenetic tree of ornithocheiroids with branch support values**

Values on nodes indicate Bremer support of nodes.


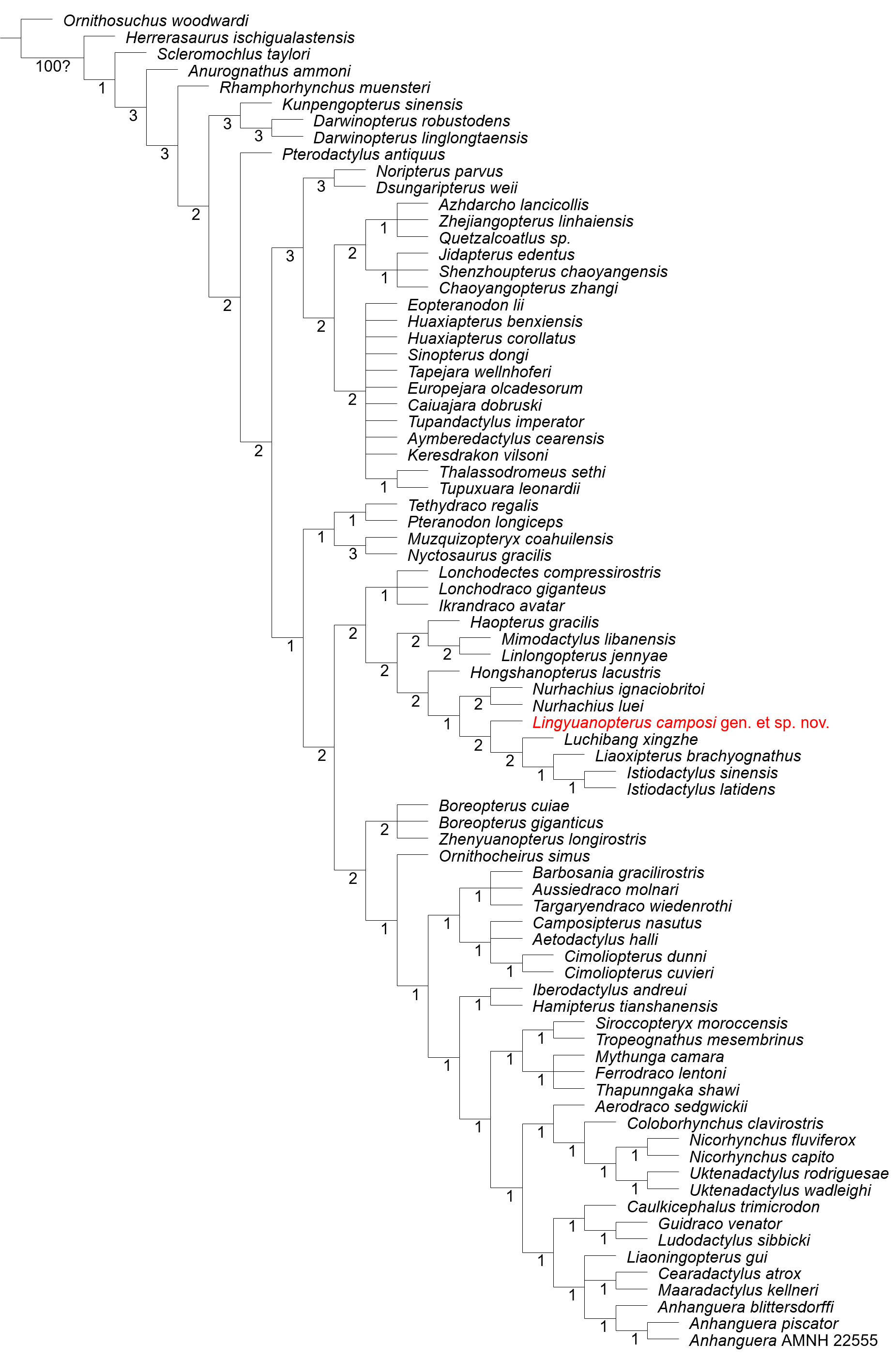

Supplement: Supplemental Information 1 [file peerj-10-13819-s001.docx]
